# Supplementary material for: Augmented temperature fluctuation aggravates muscular atrophy through the gut microbiota
Source: Nat Commun. 2023 Jun 13;14:3494. doi: 10.1038/s41467-023-39171-4 (PMC10264422; doi:10.1038/s41467-023-39171-4)
Supplement: Supplementary file 1 — Supplementary Information [file 41467_2023_39171_MOESM1_ESM.pdf]

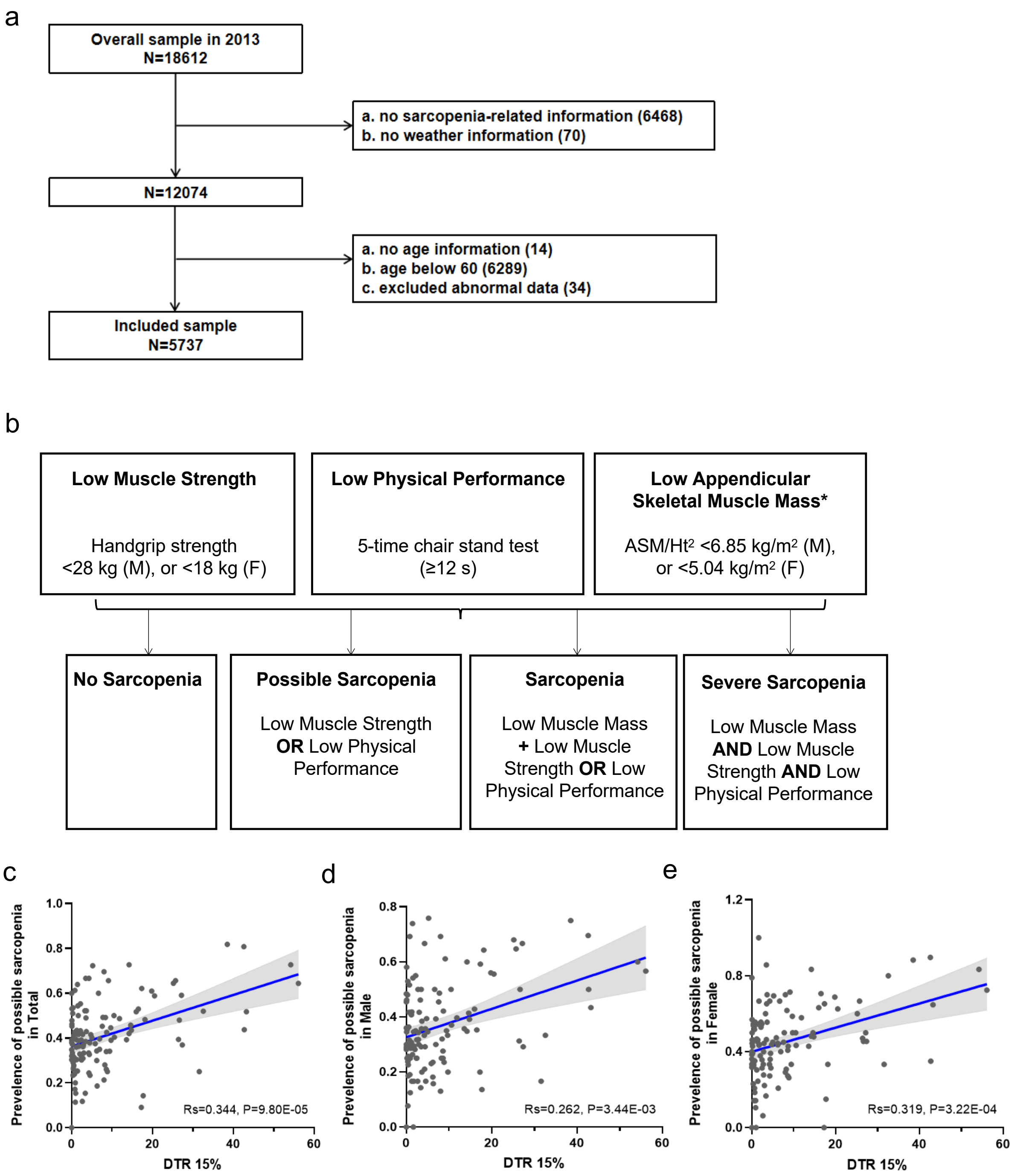
a
Overall sample in 2013
N=18612
a. no sarcopenia-related information (6468)
b. no weather information (70)
N=12074
a. no age information (14)
b. age below 60 (6289)
c. excluded abnormal data (34)
Included sample
N=5737
b
Low Muscle Strength
Handgrip strength
<28 kg (M), or <18 kg (F)
Low Physical Performance
5-time chair stand test
(≥12 s)
Low Appendicular Skeletal Muscle Mass*
ASM/Ht2 <6.85 kg/m2 (M),
or <5.04 kg/m2 (F)
No Sarcopenia
Possible Sarcopenia
Low Muscle Strength OR Low Physical Performance
Sarcopenia
Low Muscle Mass + Low Muscle Strength OR Low Physical Performance
Severe Sarcopenia
Low Muscle Mass AND Low Muscle Strength AND Low Physical Performance
c
Prevelence of possible sarcopenia in Total
DTR 15%
Rs=0.344, P=9.80E-05
d
Prevelence of possible sarcopenia in Male
DTR 15%
Rs=0.262, P=3.44E-03
e
Prevelence of possible sarcopenia in Female
DTR 15%
Rs=0.319, P=3.22E-04

Supplementary Figure 1 Data collection, diagnosis criterion and correlation analysis.

(a) Flow chart of the exclusion criteria for study population in this study.

(b) Diagnostic criteria of sarcopenia.

(c-e) Scatter plot of correlation between prevalence of possible sarcopenia and DTR 15%. DTR 15%, the proportion of days with DTR above 15°C in 2011.1.1-2013.12.31.

Data presented are mean±s.d. Spearman correlation analysis was conducted. Source data are provided as a Source Data File.

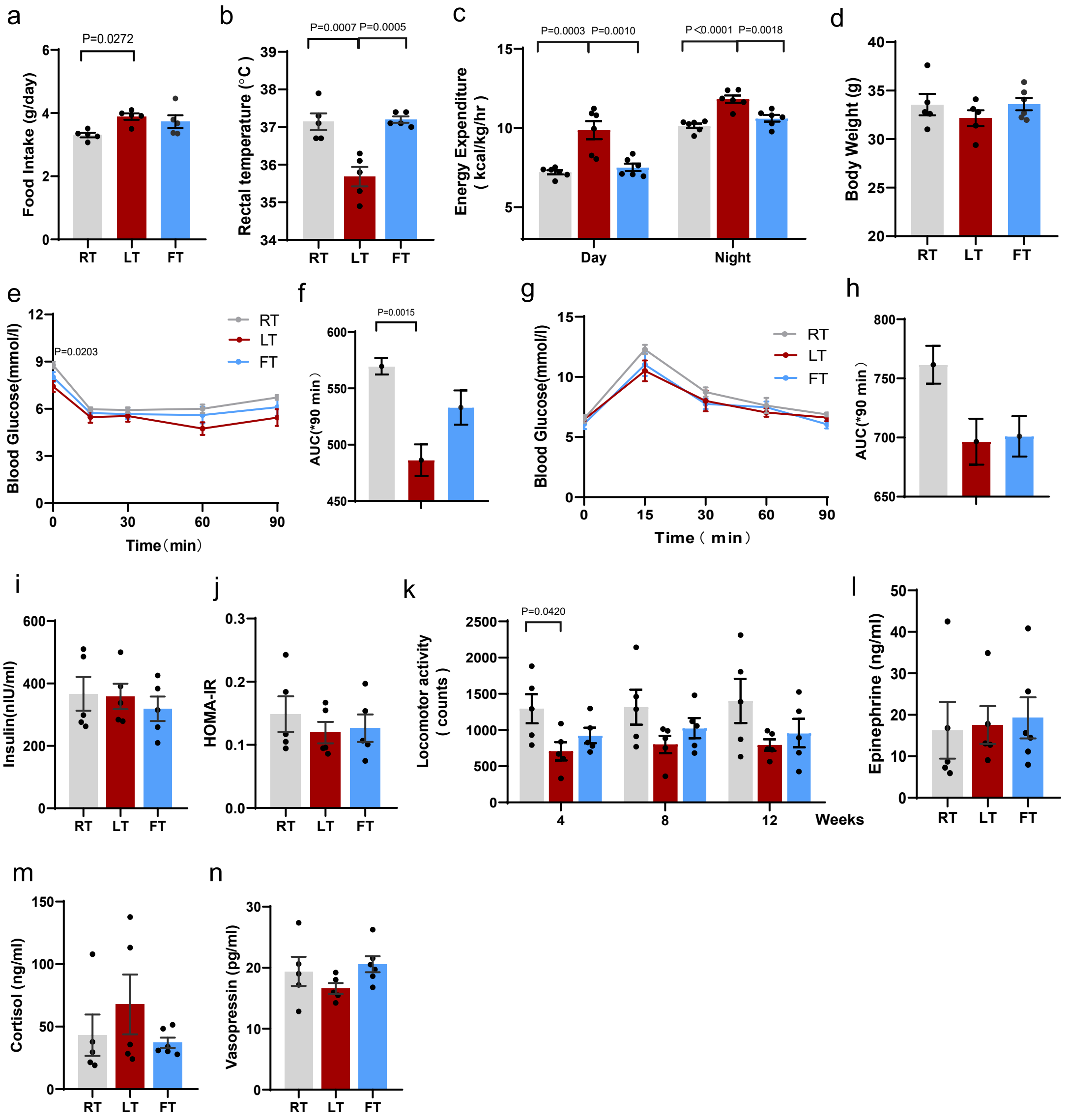
a
P=0.0272
Food Intake (g/day)
RT
LT
FT
b
P=0.0007 P=0.0005
Rectal temperature (°C)
RT
LT
FT
c
P=0.0003 P=0.0010
P <0.0001 P=0.0018
Energy Expenditure
(kcal/kg/hr)
Day
Night
d
Body Weight (g)
RT
LT
FT
e
P=0.0203
Blood Glucose(mmol/l)
Time (min)
RT
LT
FT
f
P=0.0015
AUC(*90 min)
g
Blood Glucose(mmol/l)
Time (min)
RT
LT
FT
h
AUC(*90 min)
i
Insulin(nIU/ml)
RT
LT
FT
j
HOMA-IR
RT
LT
FT
k
P=0.0420
Locomotor activity
(counts)
Weeks
l
Epinephrine (ng/ml)
RT
LT
FT
m
Cortisol (ng/ml)
RT
LT
FT
n
Vasopressin (pg/ml)
RT
LT
FT

Supplementary Figure 2 Other parameters in mice with fluctuated- temperature treatment

(a) Daily food intake of RT, LT and FT mice. n=5 biologically independent animals per group.

(b) Rectal temperature after 12-week treatment. n=5 biologically independent animals per group.

(c) Average energy expenditure. n=5 biologically independent animals per group.

(d) Body weight after 12-week treatment. n=5 biologically independent animals per group.

(e-h) ITT (e) and GTT (g) conducted after 12-week treatment, and quantification (f,h) of area under curve. n=5 biologically independent animals per group.

(i) Determination of insulin level in serum by ELISA. n=5 biologically independent animals per group.

(j) Quantification of HOMA-IR. n=5 biologically independent animals per group.

(k) Determination of locomotor activity after 4-, 8-, 12-week treatment. n=5 biologically independent animals per group.

(l-n) Determination of epinephrine, cortisol and vasopressin in serum by ELISA. n=5 biologically independent animals per group.

Data presented are mean±s.e.m. One-way ANOVA test for multiple comparisons with Tukey's test for post-hoc corrections. Source data are provided as a Source Data File.

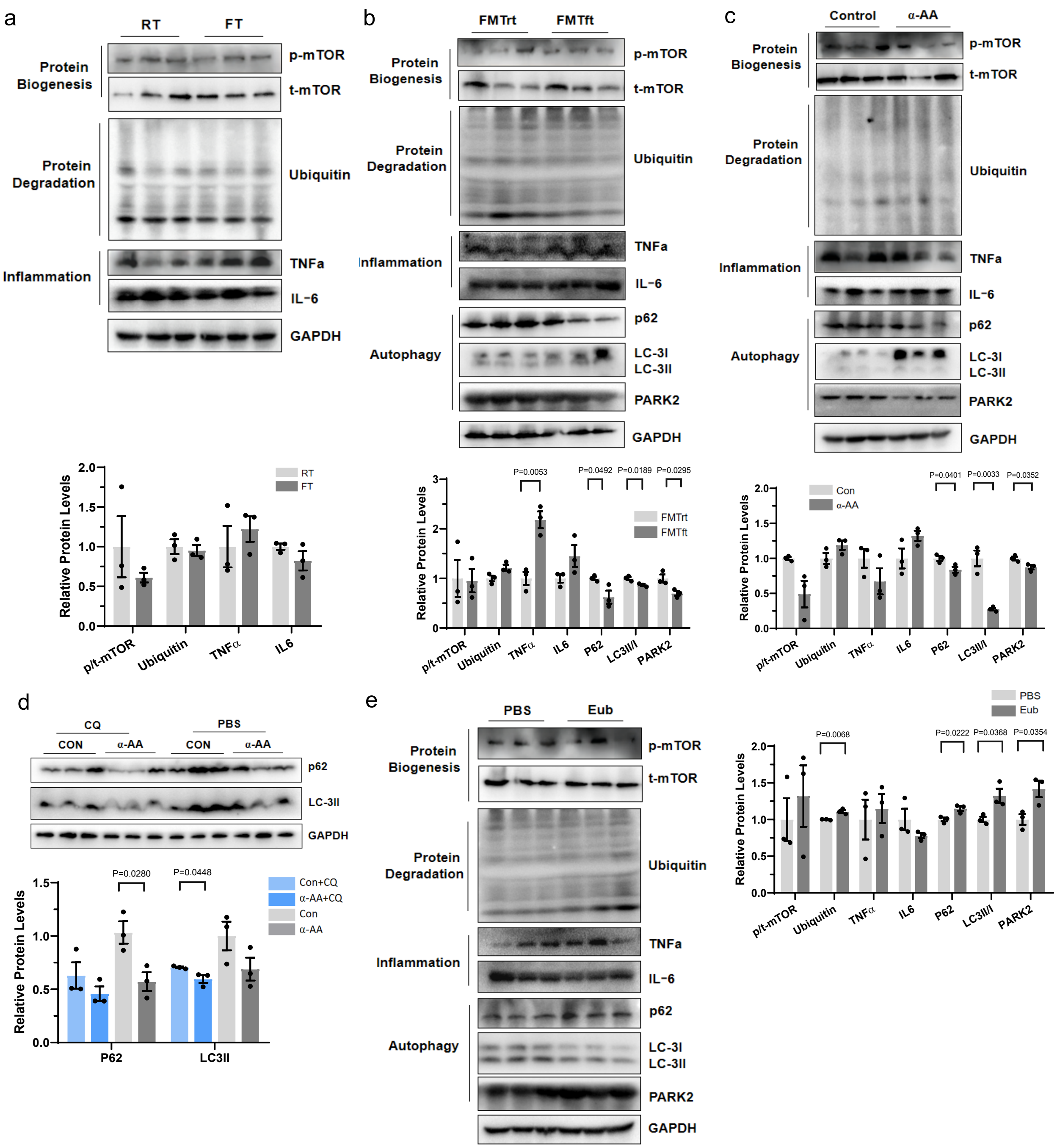

a
RT
FT
Protein Biogenesis
p-mTOR
t-mTOR
Protein Degradation
Ubiquitin
Inflammation
TNFa
IL-6
GAPDH
RT
FT
Relative Protein Levels
p/t-mTOR
Ubiquitin
TNFα
IL6
b
FMTrt
FMTft
Protein Biogenesis
p-mTOR
t-mTOR
Protein Degradation
Ubiquitin
Inflammation
TNFa
IL-6
Autophagy
p62
LC-3I
LC-3II
PARK2
GAPDH
P=0.0053
P=0.0492
P=0.0189
P=0.0295
FMTrt
FMTft
Relative Protein Levels
p/t-mTOR
Ubiquitin
TNFα
IL6
P62
LC3II/I
PARK2
c
Control
α-AA
Protein Biogenesis
p-mTOR
t-mTOR
Protein Degradation
Ubiquitin
Inflammation
TNFa
IL-6
Autophagy
p62
LC-3I
LC-3II
PARK2
GAPDH
Con
α-AA
P=0.0401
P=0.0033
P=0.0352
Relative Protein Levels
p/t-mTOR
Ubiquitin
TNFα
IL6
P62
LC3II/I
PARK2
d
CQ
PBS
CON
α-AA
CON
α-AA
p62
LC-3II
GAPDH
P=0.0280
P=0.0448
Con+CQ
α-AA+CQ
Con
α-AA
Relative Protein Levels
P62
LC3II
e
PBS
Eub
Protein Biogenesis
p-mTOR
t-mTOR
Protein Degradation
Ubiquitin
Inflammation
TNFa
IL-6
Autophagy
p62
LC-3I
LC-3II
PARK2
GAPDH
PBS
Eub
P=0.0068
P=0.0222
P=0.0368
P=0.0354
Relative Protein Levels
p/t-mTOR
Ubiquitin
TNFα
IL6
P62
LC3II/I
PARK2

Supplementary Figure 3 The expression of protein markers in mouse model

(a) Western blot (up) of protein biogenesis-, degradation-, and inflammation- related biomarkers in muscle of RT and FT mice and quantification (down) of the bands in the gels. n=3 biologically independent animals per group.

(b) Western blot (up) of protein biogenesis-, degradation-, inflammation- and autophagy- related biomarkers in muscle of FMTrt and FMTft mice and quantification (down) of the bands in the gels. n=3 biologically independent animals per group.

(c) Western blot (up) of protein biogenesis-, degradation-, inflammation- and autophagy- related biomarkers in muscle of αAA- treated mice and quantification (down) of the bands in the gels. n=3 biologically independent animals per group.

(d) Western blot (up) of autophagy markers in muscle of αAA- treated mice with or without CQ injection and quantification (down) of the bands in the gels. n=3 biologically independent animals per group.

(e) Western blot (left) of protein biogenesis-, degradation-, inflammation- and autophagy- related biomarkers in muscle of *Eubacterium*- treated mice and quantification (right) of the bands in the gels. n=3 biologically independent animals per group.

Data presented are mean±s.e.m. Two-tailed unpaired t test for binary comparison. One-way ANOVA test for multiple comparisons with Tukey's test for post-hoc corrections. Source data are provided as a Source Data File.

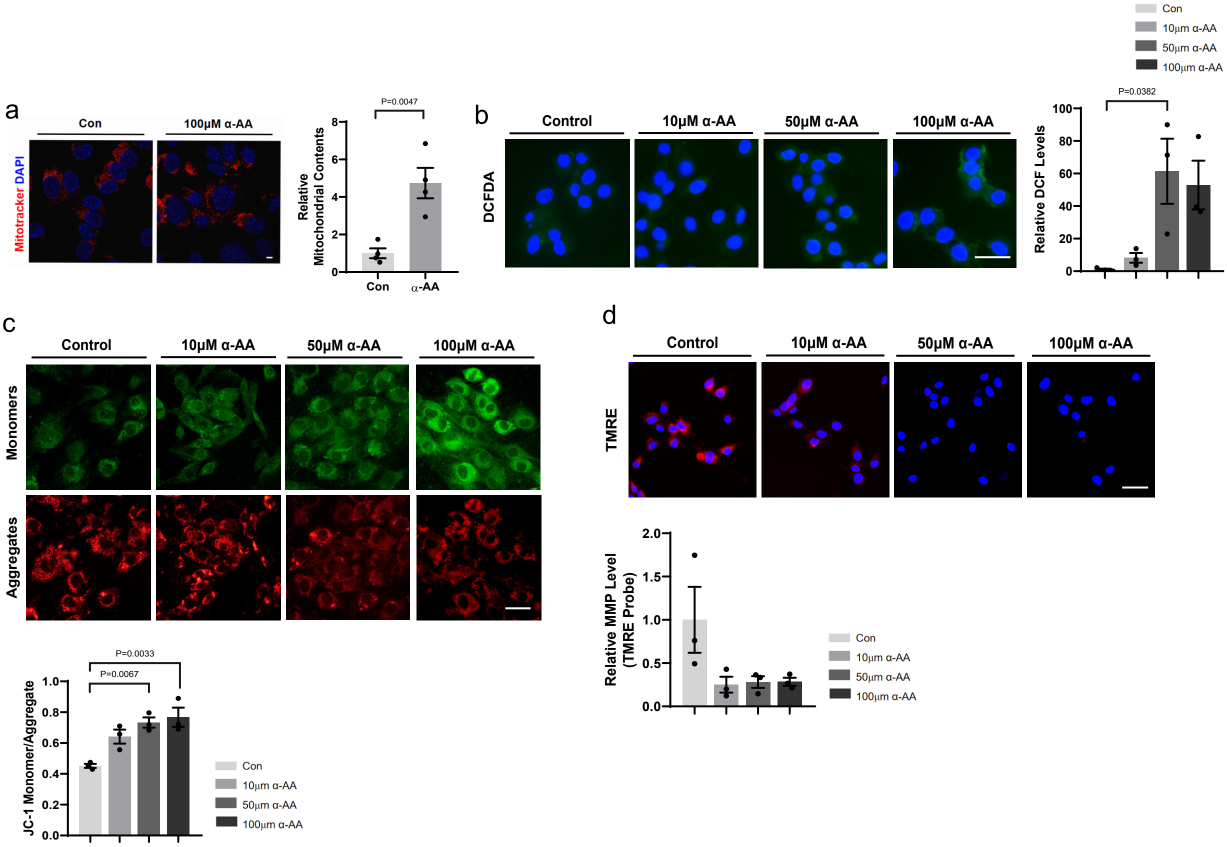

Con
10μm α-AA
50μm α-AA
100μm α-AA
a
Con
100μM α-AA
Mitotracker DAPI
P=0.0047
Relative Mitochondrial Contents
Con
α-AA
b
Control
10μM α-AA
50μM α-AA
100μM α-AA
DCFDA
P=0.0382
Relative DCF Levels
c
Control
10μM α-AA
50μM α-AA
100μM α-AA
Monomers
Aggregates
P=0.0033
P=0.0067
JC-1 Monomer/Aggregate
Con
10μm α-AA
50μm α-AA
100μm α-AA
d
Control
10μM α-AA
50μM α-AA
100μM α-AA
TMRE
Relative MMP Level (TMRE Probe)
Con
10μm α-AA
50μm α-AA
100μm α-AA

Supplementary Figure 4 Effects of α-AA treatment on mitochondria in C2C12

(a) Representative images of mitochondrial staining (left) by Mitotracker, and quantification (right) of stained area. Bar=10μm. n=4 biologically independent cells per group.

(b) Representative images of DCF probe level (left) after α-AA treatment, and quantification (right) of fluorescence intensity. Scale bar=30μm. n=3 biologically independent cells per group.

(c) Representative images of JC-1 probe level (up) after α-AA treatment, and quantification (down) of fluorescence intensity. Scale bar=40μm. n=3 biologically independent cells per group.

(d) Representative images of TMRE probe level (up) after α-AA treatment, and quantification (down) of fluorescence intensity. Scale bar=40μm. n=3 biologically independent cells per group.

Data presented are mean±s.e.m. Two-tailed unpaired t test for binary comparison. One-way ANOVA test for multiple comparisons with Tukey's test for post-hoc corrections. Source data are provided as a Source Data File.

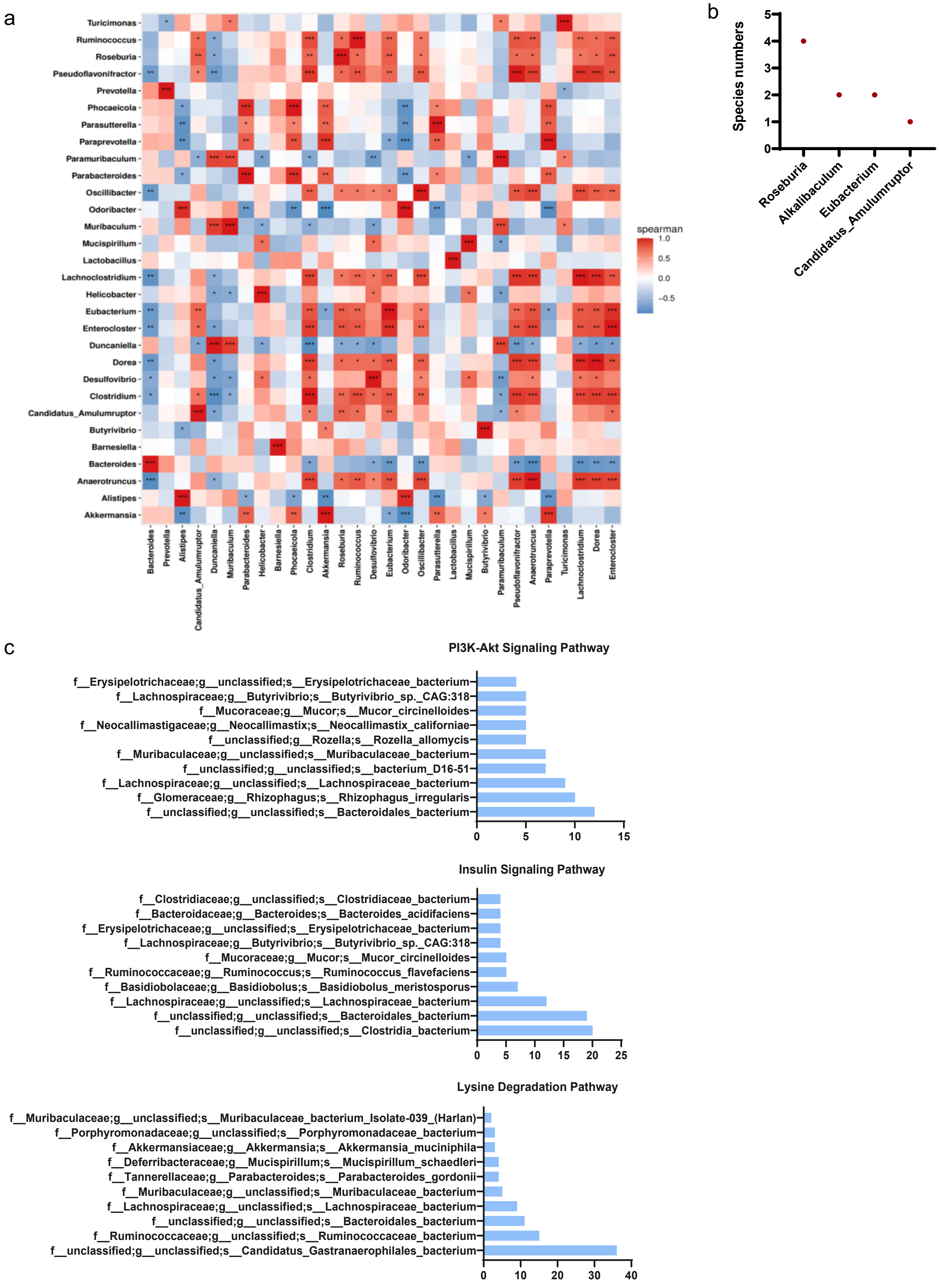
a
b
c
spearman
Species numbers
Roseburia
Alkalibaculum
Eubacterium
Candidatus_Amulumruptor
PI3K-Akt Signaling Pathway
f__Erysipelotrichaceae;g__unclassified;s__Erysipelotrichaceae_bacterium
f__Lachnospiraceae;g__Butyrivibrio;s__Butyrivibrio_sp._CAG:318
f__Mucoraceae;g__Mucor;s__Mucor_circinelloides
f__Neocallimastigaceae;g__Neocallimastix;s__Neocallimastix_californiae
f__unclassified;g__Rozella;s__Rozella_allomycis
f__Muribaculaceae;g__unclassified;s__Muribaculaceae_bacterium
f__unclassified;g__unclassified;s__bacterium_D16-51
f__Lachnospiraceae;g__unclassified;s__Lachnospiraceae_bacterium
f__Glomeraceae;g__Rhizophagus;s__Rhizophagus_irregularis
f__unclassified;g__unclassified;s__Bacteroidales_bacterium
Insulin Signaling Pathway
f__Clostridiaceae;g__unclassified;s__Clostridiaceae_bacterium
f__Bacteroidaceae;g__Bacteroides;s__Bacteroides_acidifaciens
f__Erysipelotrichaceae;g__unclassified;s__Erysipelotrichaceae_bacterium
f__Lachnospiraceae;g__Butyrivibrio;s__Butyrivibrio_sp._CAG:318
f__Mucoraceae;g__Mucor;s__Mucor_circinelloides
f__Ruminococcaceae;g__Ruminococcus;s__Ruminococcus_flavefaciens
f__Basidiobolaceae;g__Basidiobolus;s__Basidiobolus_meristosporus
f__Lachnospiraceae;g__unclassified;s__Lachnospiraceae_bacterium
f__unclassified;g__unclassified;s__Bacteroidales_bacterium
f__unclassified;g__unclassified;s__Clostridia_bacterium
Lysine Degradation Pathway
f__Muribaculaceae;g__unclassified;s__Muribaculaceae_bacterium_Isolate-039_(Harlan)
f__Porphyromonadaceae;g__unclassified;s__Porphyromonadaceae_bacterium
f__Akkermansiaceae;g__Akkermansia;s__Akkermansia_muciniphila
f__Deferribacteraceae;g__Mucispirillum;s__Mucispirillum_schaedleri
f__Tannerellaceae;g__Parabacteroides;s__Parabacteroides_gordonii
f__Muribaculaceae;g__unclassified;s__Muribaculaceae_bacterium
f__Lachnospiraceae;g__unclassified;s__Lachnospiraceae_bacterium
f__unclassified;g__unclassified;s__Bacteroidales_bacterium
f__Ruminococcaceae;g__unclassified;s__Ruminococcaceae_bacterium
f__unclassified;g__unclassified;s__Candidatus_Gastranaerophilales_bacterium

Supplementary Figure 5 Data analysis based on shotgun metagenomics

(a) Correlation analysis of bacteria with top 30 in total abundance in genus level.

(b) Decreased species in FT mice contributing to lysine degradation pathway.

(c) Species contributing to KEGG pathway with high LDA score in FT group. (Top 10 were shown)

Supplementary Table 1

List of oligonucleotide primer pairs used in RT-PCR analysis

Target Gene	Sense Primer	Antisense Primer
mt-Co2	5'-GTTGATAACCGAGTCGTTCTGC-3'	5'-CCTGGGATGGCATCAGTTTT-3'
mt-Rnr2	5'-CCGCAAGGGAAAGATGAAAGAC-3'	5'-TCGTTTGGTTTCGGGGTTTC-3'
Ucp2	5'-CTACAGATGTGGTAAAGGTCCGC-3'	5'-GCAATGGTCTTGTAGGCTTCG-3'
Hk2	5'-TCTGGCTCTGAGATCCATCTTCA-3'	5'-CCGGCCTCTTAACCACATTCC-3'
Nrf1	5'-AGCACGGAGTGACCCAAAC-3'	5'-TGTACGTGGCTACATGGACCT-3'
Tfam	5'-ATTCCGAAGTGTTTTTCCAGCA-3'	5'-TCTGAAAGTTTTGCATCTGGGT-3'
Ppargc-1α	5'-CCCTGCCATTGTTAAGACC-3'	5'-TGCTGCTGTTCCTGTTTTC-3'
Mfn1	5'-CCTACTGCTCCTTCTAACCCA-3'	5'-AGGGACGCCAATCCTGTGA-3'
Dnm1l	5'-TTACGGTTCCCTAAACTTCACG-3'	5'-GTCACGGGCAACCTTTTACGA-3'
Opa1	5'-TGGAAAATGGTTCGAGAGTCAG-3'	5'-CATTCCGTCTCTAGGTTAAAGCG-3'
Fis1	5'-TGTCCAAGAGCACGCAATTTG-3'	5'-CCTCGCACATACTTTAGAGCCTT-3'
Myostatin	5'-AGTGGATCTAAATGAGGGCAGT-3'	5'-GTTTCCAGGCGCAGCTTAC-3'
Atrogin	5'-CAGAGAGGCAGATTCGCAAG-3'	5'-GGTGACCCCATACTGCTCTC-3'
Trim63	5'-CATCTTCCAGGCTGCGAATC-3'	5'-ACTGGAGCACTCCTGCTTGT-3'
Musa1	5'-CTTCAGTCTCGTGGAATGGTAATCTT-3'	5'-TGCAGTACTGAATCGCCATAC-3'
Becn1	5'-CCGCGGTAGAACGAGCC-3'	5'-AAGTAATGGAGCTGTGAGTTCCT-3'
Pik3c3	5'-GTGAAGTACCCTGACCTGCC-3'	5'-AGTCATGCATTCCTTGGCGA-3'
Atg5	5'-GGAGAGAAGAGGAGCCAGGT -3'	5'-GCTGGGGGACAATGCTAATA-3'
p62	5'-GCTGAAGGAAGCTGCCCTAT-3'	5'-TTGGTCTGTAGGAGCCTGGT-3'
Lc3b	5'-CACTGCTCTGTCTTGTGTAGGTTG-3'	5'-TCGTTGTGCCTTTATTAGTGCATC-3'
Park2	5'-CCGAATCACCTGACGGTTCA-3'	5'-TCTGGCTGCTTCTGAATCCC-3'

Supplementary Table 2

Mean DTR in 2011.1.1-2013.12.31

Province	City	Mean DTR (℃)	Province	City	Mean DTR (℃)
Anhui	Anqing	8.32	Jiangsu	Yancheng	8.59
	Bozhou	9.60		Yangzhou	8.88
	Fuyang	10.09	Jiangxi	Nanchang	7.35
	Huainan	8.35		Ganzhou	8.44
	Liuan	9.18		Jian	8.49
	Suzhou	9.42		Jingdezhen	9.26
	Beijing	9.88		Jiujiang	7.65
Chongqing	Chongqing	7.26		Shangrao	9.42
Fujian	Fuzhou	7.78		Yichun	8.68
	Ningde	7.17	Liaoning	Anshan	8.26
	Putian	6.78		Benxi	10.25
	Zhangzhou	8.06		Chaoyang	12.43
Gansu	Lanzhou	13.27		Dalian	6.25
	Dingxi	12.50		Jinzhou	9.65
	Pingliang	11.89	Neimenggu	Hohhot	11.93
	Zhangye	15.55		Chifeng	11.88
Guangdong	Guangzhou	7.79		Hulunbuir	13.26
	Chaozhou	8.47		Hinggan	10.88
	Maoming	7.56		Xilingol	12.91
	Qingyuan	7.16	Qinghai	Haidong	14.24
	Jiangmen	6.57		Jinan	9.11
	Shenzhen	6.02		Binzhou	10.65
	Foshan	6.43	Shandong	Dezhou	9.66
Guangxi	Nanning	7.88		Liaocheng	10.50
	Yulin	7.47		Linyi	10.00
	Guilin	7.19		Qingdao	5.99
	Hechi	7.74		Weihai	6.56
Guizhou	Qiannan	7.84		Weifang	10.41
	Qiandongnan	9.06		Zaozhuang	9.62
Hebei	Shijiazhuang	8.78	Shanxi	Linfen	11.45
	Baoding	10.91		Xinzhou	13.70
	Cangzhou	10.25		Yangquan	12.00
	Chengde	13.25		Yuncheng	11.74

Henan	Zhengzhou	9.98	Shanghai	Shanghai	7.39
	Anyang	10.77	Shaanxi	Baoji	10.36
	Jiaozuo	10.31		Hanzhong	8.79
	Luoyang	9.88		Weinan	10.77
	Pingdingshan	10.18		Yulin	11.86
	Puyang	10.59	Sichuan	Chengdu	8.78
	Xinyang	8.86		Ganzi	15.01
	Zhoukou	9.47		Guangan	7.39
Heilongjiang	Harbin	9.75		Nanchong	7.68
	Jixi	9.73		Liangshan	11.90
	Jiamusi	10.48		Mianyang	7.90
	Qiqihar	10.12		Neijiang	7.78
Hubei	Enshi	8.32		Yibin	7.49
	Huanggang	8.16		Ziyang	7.86
	Jingmen	8.18		Meishan	7.91
	Xiangfan	8.80	Tianjin	Tianjin	9.82
Hunan	Changsha	7.42	Xinjiang	Akesu	12.90
	Changde	8.56	Yunnan	Kunming	11.05
	Loudi	8.51		Baoshan	11.63
	Shaoyang	8.45		Chuxiong	11.01
	Yiyang	7.94		Lijiang	12.24
	Yueyang	6.45		Lincang	12.28
Jilin	Jilin	9.27		Zhaotong	9.93
	Siping	9.93	Zhejiang	Hangzhou	8.42
Jiangsu	Lianyungang	9.33		Huzhou	8.84
	Suzhou	7.91		Jiaxing	8.13
	Suqian	8.87		Lishui	10.21
	Taizhou	8.65		Ningbo	8.56
	Xuzhou	9.54		Taizhou	7.56
