# Supplementary material for: Augmented temperature fluctuation aggravates muscular atrophy through the gut microbiota
Source: Nat Commun. 2023 Jun 13;14:3494. doi: 10.1038/s41467-023-39171-4 (PMC10264422; doi:10.1038/s41467-023-39171-4)
Supplement: Supplementary file 3 — Reporting Summary [file 41467_2023_39171_MOESM3_ESM.pdf]

nature portfolio

Corresponding author(s): Yan Huang

Last updated by author(s): April 4, 2023

Reporting Summary

Nature Portfolio wishes to improve the reproducibility of the work that we publish. This form provides structure for consistency and transparency in reporting. For further information on Nature Portfolio policies, see our Editorial Policies and the Editorial Policy Checklist.

Statistics

For all statistical analyses, confirm that the following items are present in the figure legend, table legend, main text, or Methods section.

n/a	Confirmed	
☐	☒	The exact sample size (*n*) for each experimental group/condition, given as a discrete number and unit of measurement
☐	☒	A statement on whether measurements were taken from distinct samples or whether the same sample was measured repeatedly
☐	☒	The statistical test(s) used AND whether they are one- or two-sided<br>*Only common tests should be described solely by name; describe more complex techniques in the Methods section.*
☒	☐	A description of all covariates tested
☒	☐	A description of any assumptions or corrections, such as tests of normality and adjustment for multiple comparisons
☐	☒	A full description of the statistical parameters including central tendency (e.g. means) or other basic estimates (e.g. regression coefficient) AND variation (e.g. standard deviation) or associated estimates of uncertainty (e.g. confidence intervals)
☐	☒	For null hypothesis testing, the test statistic (e.g. *F*, *t*, *r*) with confidence intervals, effect sizes, degrees of freedom and *P* value noted<br>*Give P values as exact values whenever suitable.*
☒	☐	For Bayesian analysis, information on the choice of priors and Markov chain Monte Carlo settings
☒	☐	For hierarchical and complex designs, identification of the appropriate level for tests and full reporting of outcomes
☒	☐	Estimates of effect sizes (e.g. Cohen's *d*, Pearson's *r*), indicating how they were calculated

Our web collection on statistics for biologists contains articles on many of the points above.

Software and code

Policy information about availability of computer code

Data collection	No software were use for data collection.
Data analysis	GraphPad Prism 7 (statistical analysis), SPSS 21 (correlation analysis), QIIME software Ver 1.8.0 (16S rRNA), DIAMOND software V0.9.7 (Metagenome) .

For manuscripts utilizing custom algorithms or software that are central to the research but not yet described in published literature, software must be made available to editors and reviewers. We strongly encourage code deposition in a community repository (e.g. GitHub). See the Nature Portfolio guidelines for submitting code & software for further information.

Data

Policy information about availability of data

All manuscripts must include a data availability statement. This statement should provide the following information, where applicable:

- Accession codes, unique identifiers, or web links for publicly available datasets
- A description of any restrictions on data availability
- For clinical datasets or third party data, please ensure that the statement adheres to our policy

We have included data availability statement in our manuscript. The data supporting the findings are available from the corresponding author on reasonable request. Raw data of figures are provided in Source Data file.

Human research participants

Policy information about studies involving human research participants and Sex and Gender in Research.

Reporting on sex and gender	We used human CHARLS database 2013. Both gender were included in this study. Source data can be downloaded form CHARLS official website in individual level. 5737 participants were enrolled for analyze. The following correlation analysis were conducted in sex-based way.
Population characteristics	Participants were categorized into three groups, including No sarcopenia (NS), Possible sarcopenia(PS) and Sarcopenia(S), based on the diagnostic criteria of AWGS 2019. Age of participants with NS, PS and S were 66.23±5.36, 68.64±6.63, 73.44 ±7.21. BMI of male participants with NS, PS and S were 23.18±6.19, 24.45±9.98 and 18.36±1.74, and BMI of female participants with NS, PS and S were 24.15±6.35, 25.3±3.62 and 18.94±1.87.
Recruitment	Total of 18,612 participants were screened from CHARLS database 2013. Among them, 6542 participants were excluded for missing necessary information, 6289 of them were excluded for age, 34 of them were excluded for abnormal data. 5737 out of 18,612 participants were recruited for further study.
Ethics oversight	CHARLS datasets are open to public and can be downloaded from CHARLS official website (http://charls.pku.edu.cn/en). Ethical approval for all the CHARLS waves was granted from the Institutional Review Board at Peking University, and all participants signed informed consent.

Note that full information on the approval of the study protocol must also be provided in the manuscript.

Field-specific reporting

Please select the one below that is the best fit for your research. If you are not sure, read the appropriate sections before making your selection.

☒ Life sciences ☐ Behavioural & social sciences ☐ Ecological, evolutionary & environmental sciences

For a reference copy of the document with all sections, see nature.com/documents/nr-reporting-summary-flat.pdf

Life sciences study design

All studies must disclose on these points even when the disclosure is negative.

Sample size	Sample size was based on existing published literature of similar experiments. For animal experiments, n≥5 was chosen based on the previous publications in the field (Li, C.J., et al., 2021, Cell Metab). For cell experiments, n≥3 was chosen based on the previous publications in the field (Peng, H., et al., 2022, Cell metabolism) and also because this size is necessary to calculate statistical significances.
Data exclusions	No initial exclusion criteria were used for both in vitro and in vivo experiments.
Replication	All experimental findings were reliably reproduced for three times and all replication attempts were successful.
Randomization	All the samples were randomly assigned.
Blinding	None of the experiments in the study were performed in a blinded fashion. And investigators were blinded during data analysis.

Reporting for specific materials, systems and methods

We require information from authors about some types of materials, experimental systems and methods used in many studies. Here, indicate whether each material, system or method listed is relevant to your study. If you are not sure if a list item applies to your research, read the appropriate section before selecting a response.

Materials & experimental systems

n/a	Involved in the study	
☐	☒	Antibodies
☐	☒	Eukaryotic cell lines
☒	☐	Palaeontology and archaeology
☐	☒	Animals and other organisms
☒	☐	Clinical data
☒	☐	Dual use research of concern

Methods

n/a	Involved in the study	
☒	☐	ChIP-seq
☒	☐	Flow cytometry
☒	☐	MRI-based neuroimaging

Antibodies

Antibodies used	anti-P62 (CST, 5114), anti-LC3B (CST, 2775), anti-PARK2 (Proteintech, 14060-1-AP), anti-Ubiquitin (CST, 3933), anti-TFAM (Santa Cruz, sc-166965), anti-PGC1a (Santa Cruz, sc-518025), anti-MFN1 (Santa Cruz, sc-166644), anti-OPA1 (Santa Cruz, sc-393296), anti-phospho-mTOR (CST, 5536), anti-mTOR (CST, 2983), anti-TNFα (Santa Cruz, sc-52746), anti-IL6 (Santa Cruz, sc-28343), anti-GAPDH (OriGene, TA802519). Secondary antibodies (Invitrogen, 31430 and 31460).
Validation	anti-P62 (CST, 5114): Mouse, WB; anti-LC3B (CST, 2775): Mouse, WB; anti-PARK2 (Proteintech, 14060-1-AP): Mouse, WB; anti-Ubiquitin (CST, 3933): Mouse, WB; anti-TFAM (Santa Cruz, sc-166965): Mouse, WB; anti-PGC1a (Santa Cruz, sc-518025): Mouse, WB; anti-MFN1 (Santa Cruz, sc-166644): Mouse, WB; anti-OPA1 (Santa Cruz, sc-393296): Mouse, WB; anti-phospho-mTOR (CST, 5536): Mouse, WB; anti-mTOR (CST, 2983): Mouse, WB; anti-TNFα (Santa Cruz, sc-52746): Mouse, WB; anti-IL6 (Santa Cruz, sc-28343): Mouse, WB; anti-GAPDH (OriGene, TA802519): Mouse, WB. secondary antibodies (Invitrogen, 31430): GAM,WB; secondary antibodies (Invitrogen, 31460): GAR, WB.

Eukaryotic cell lines

Policy information about cell lines and Sex and Gender in Research

Cell line source(s)	C2C12 cell line were purchased from Procell Co (China).
Authentication	STR authentication report were provided.
Mycoplasma contamination	Cell lines were not tested for mycoplasma contamination.
Commonly misidentified lines (See ICLAC register)	No commonly misidentified cell line was used.

Animals and other research organisms

Policy information about studies involving animals; ARRIVE guidelines recommended for reporting animal research, and Sex and Gender in Research

Laboratory animals	12-month-old male C57BL/6J mice were used in all experiments. Except for mouse model of fluctuated temperature treatment, other mice were kept at a controlled temperature (23-25°C) and humidity (40%), with 12-h day/night cycle and fed a standard chow diet ad libitum.
Wild animals	The study didn't involve wild animals.
Reporting on sex	Only male mice were used for experiments.
Field-collected samples	The study didn't involve sample from field.
Ethics oversight	Animal experiments were approved by the Animal Care and Use Committees of the Laboratory Animal Research Center at Xiangya Medical School of Central South University.

Note that full information on the approval of the study protocol must also be provided in the manuscript.
